# The global burden of urinary bladder cancer: an update

**DOI:** 10.1007/s00345-019-02984-4

**Published:** 2019-11-01

**Authors:** Anke Richters, Katja K. H. Aben, Lambertus A. L. M. Kiemeney

**Affiliations:** 1 Netherlands Comprehensive Cancer Organisation, Utrecht, The Netherlands; 2 Radboud Institute for Health Sciences, Radboud university medical center, Nijmegen, The Netherlands

**Keywords:** Urinary bladder cancer, Incidence, Prevalence, Mortality, Global burden

## Abstract

Bladder cancer is among the top ten most common cancer types in the world, with approximately 550,000 new cases annually. The highest burden of bladder cancer is currently falling on most developed communities across the globe. But with an anticipated shift in world demographics with growing and aging populations mainly on the African continent, and important shifts in exposure to different risk factors across the world, this is likely to change over the next decades. In this review, we provide an overview of the current incidence, mortality, prevalence, survival, risk factors and costs of bladder cancer worldwide.

## Cancer across the globe

Each year, more than 18 million new cases of cancer are diagnosed worldwide. Globally, one in every five people will develop the disease before the age of 75 years, but this varies from 1 in 10 in South-Central Asia and Middle-Africa to 1 in 2.5 in Australia and New Zealand. Currently, there are approximately 44 million cancer patients alive who were diagnosed less than 5 years ago. Almost 10 million people die from cancer annually [1].

Approximately 3.0% of all new cancer diagnoses and 2.1% of all cancer deaths are due to urinary bladder cancer (UBC) [1]. In this overview we will describe the burden of UBC in the world regarding incidence, survival, mortality, prevalence and risk factors. Moreover, we reflect on (anticipated) changes in demography and economic situation in different parts of the world and the impact these changes may have on the burden of UBC [2].

## Assessing the burden of UBC is complicated by definition differences

Assessing the burden of UBC worldwide is reliant upon the availability of cancer registry data [3]. The International Association of Cancer Registries (IACR) has made major steps towards increasing the proportion of the world population covered by national or regional cancer registries. To date, national cancer registries are mainly situated in European countries and Australia. In other, more developed countries, often regional cancer registries are present whereas in less developed countries there is a lack of national and/or regional population-based cancer registries [4, 5].

Several international rules and guidelines have been developed to enhance comparability across cancer registries. In general, cancer registries adhere to the International Classification of Diseases for Oncology (ICD-O) and other systems such as the TNM system and grading systems.

Despite the use of uniform coding systems like the ICD-O and TNM, UBC has some specific features that should be kept in mind when comparing data from different areas of the world or different time periods. Some cancer registries include non-invasive (Tis and Ta) UBC while other registries only include invasive (≥ T1) cancers. T1 tumors are invasive, but not muscle-invasive. Their clinical course and treatment are more like Ta tumors, which is reason to group Ta and T1 tumors as non-muscle-invasive tumors. Because Ta tumors make up about 50% of all new UBC diagnoses, inclusion or exclusion of these Ta tumors has an enormous impact. While non-muscle-invasive tumors are usually treated locally, treatment of muscle-invasive tumors often involves a radical cystectomy or alternatively, (chemo)radiation and have much worse survival rates. Naturally, such registration differences may have a huge effect on the comparison of incidence, prevalence and survival estimates.

In addition to registration differences, the term ‘invasive’ may be confusing in reports or publications if it is not well-defined because it may both be used to describe cancers that invade the lamina propria (≥ T1, as defined by the TNM system), or tumors that invade the bladder muscle (≥ T2, as usually referred to by clinicians).

In this paper, most data are derived from GLOBOCAN 2018 which includes non-invasive UBC, unless stated otherwise. GLOBOCAN (The Global Cancer Observatory by the International Agency for Research on Cancer [IARC]) is based on several key IACR projects, including Cancer Incidence in Five Continents (CI5), and offers an interactive web-based platform presenting global cancer statistics. It includes a module that provides data visualization tools that present current national estimates of the incidence, mortality and prevalence of 36 cancer types in 185 countries by sex and age group [6].

## UBC occurrence is about threefold higher in Europe and North America

**Fig. 1:**
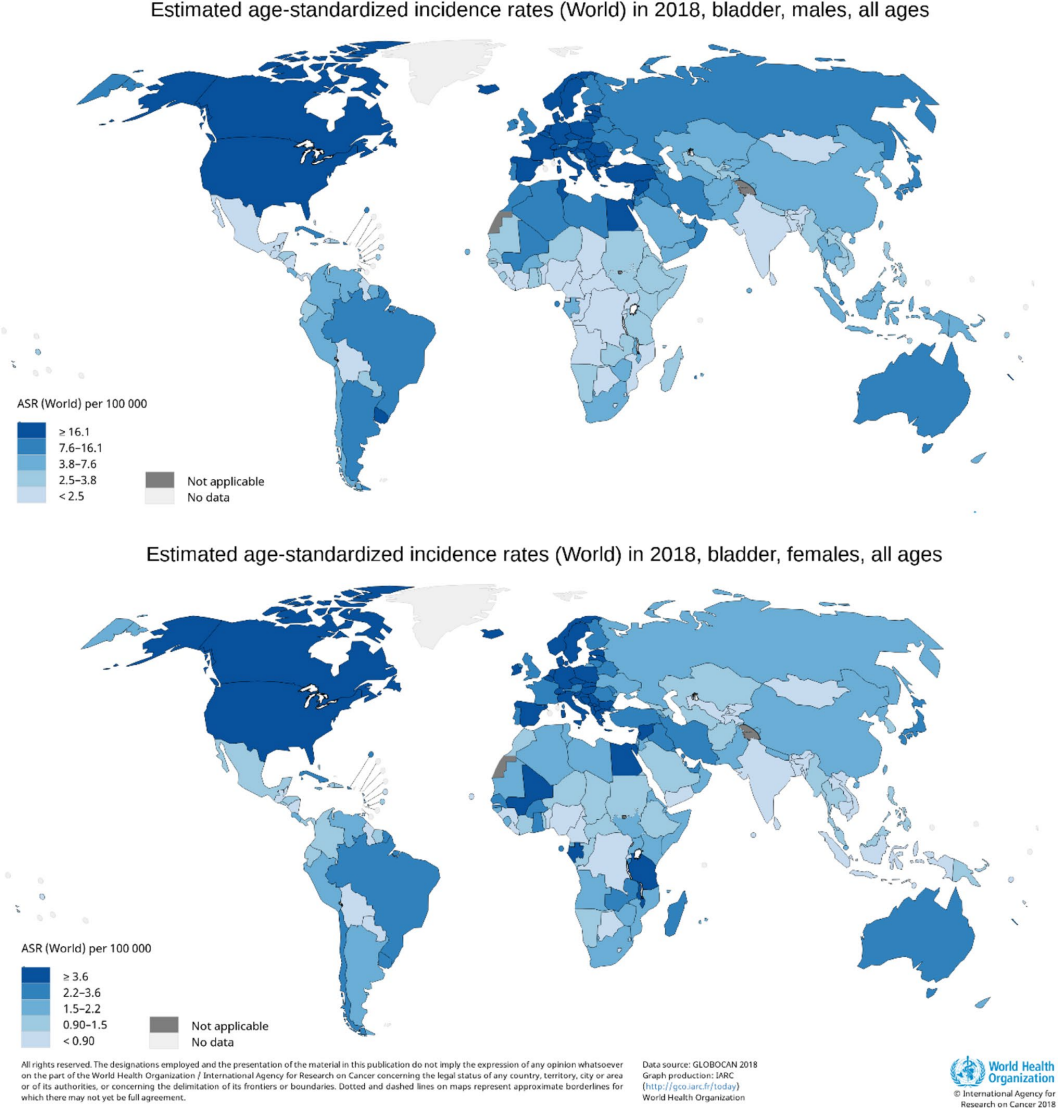
Urinary bladder cancer incidence in 2018 in the world, by sex

Although usually not perceived as such by the general population, UBC is among the more commonly occurring cancers. It ranks tenth in worldwide absolute incidence: sixth in men and seventeenth in women [6]. Approximately 550,000 new UBCs (almost 425,000 in males and over 125,000 in females) were diagnosed worldwide in 2018 [6]. The worldwide Age Standardized Incidence Rate per year (ASR) is 9.6 per 100,000 for males and 2.4 per 100,000 for females. Figure 1 shows the worldwide ASRs for UBC in both sexes.

The incidence varies significantly between geographical regions, with the highest rates (ASR in males ≈ 20 per 100,000 per year and ASR in females ≈ 4.5 per 100,000 per year) observed in Europe and North America, but also in Syrian, Israeli, Egyptian and Turkish males. About threefold lower rates are seen in South-East Asia, except for Japan, and in Latin America and Northern Africa in both sexes [4]. The lowest rates are observed in Sub-Saharan Africa, Mexico and some Middle Eastern and Central Asian countries.

Worldwide differences in (historical) exposure to risk factors like cigarette smoking, chemical carcinogens in certain occupations and arsenic in drinking water or endemic chronic urinary infections caused by *Schistosoma haematobium* are largely responsible for the observed variability in occurrence, although a small part of the geographical differences may also be attributable to differences in access to care and availability of diagnostic procedures such as enhanced cystoscopy (e.g. narrow band imaging or blue light cystoscopy) and computed tomography between more and less developed countries [7]. Cigarette smoking is by far the number one risk factor, accounting for up to 50% of all UBC diagnoses, especially urothelial cell cancer (UCC) [8]. Schistosomiasis is mainly associated with the development of squamous cell carcinoma (SCC). In regions where this infection is or was endemic, specifically Egypt, SCC was for years the dominant histopathological type of UBC. However, changes occurred over the past decades in these regions with increasing proportions of UC, while SCC decreased from 78% in 1980 to 27% in 2005 [9]. This is probably caused by public health interventions and the construction of the Aswan Higher Dam in the Nile Delta in the 1960s. This led to a replacement of *S. haematobium* with *S. mansoni*, which is not a risk factor for UBC. The prevalence of *S. haematobium* infection reduced from 74% in 1935 to only 4% in 1983 [10]. The extremely high UBC incidence (ASR for both sexes > 20 per 100,000) in Lebanon and Greece may still reflect exposure to *S. haematobium*, but is expected to decrease due to efforts by the WHO on preventive treatment for schistosomiasis worldwide, especially in school-aged children, negating the long-term effects of the infection [11].

## About 1 in 100 men and 1 in 400 women will be diagnosed with UBC sometime in life worldwide

**Fig. 2:**
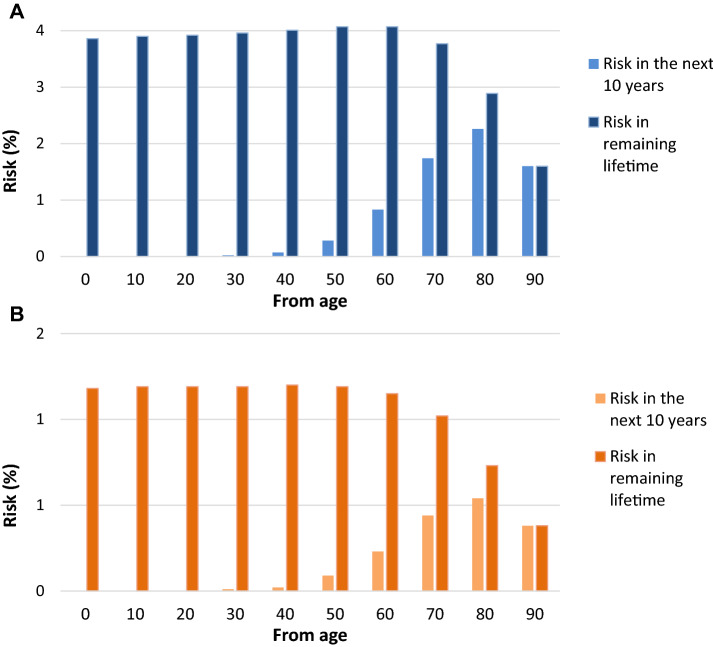
Risk of developing urinary bladder cancer for males (**a**) and females (**b**) in the US. Based on data from the Surveillance, Epidemiology, and End Results Program (SEER), US, 2014–2016, all races, males and females [12]. Examples of interpretation: a 50-year old man has an average risk of 0.28% to be diagnosed with bladder cancer before his 60th birthday. A 60-year old woman has an average risk of 1.15% to be diagnosed with bladder cancer in the remainder of her life

Worldwide, the lifetime risk of getting UBC is 1.1% in men, and 0.27% in women [6]. The lifetime risk is considerably higher in most developed countries, such as the US (lifetime risk in men and women of 3.9% and 1.2%, respectively) [12]. Figure 2 shows that the lifetime risk among men and women in the US slightly increases from birth until the age of 50 years. Only after that, the risk to be diagnosed in the remaining life years starts to decline [12]. The risk of developing UBC in the next 10 years is also presented in Fig. 2 for men and women of different ages. This risk is highest in 80-year olds in both males and females.

It should be noted that the lifetime risks are derived from current incidence rates of UBC, reflecting UBC predominantly diagnosed among the elderly, and their past exposure to risk factors. If the prevalence of these risk factors has changed over time, the actual lifetime risks of the younger cohorts will deviate from the risks reported here.

In Table 1 the risk of developing UBC from and until a certain age is presented for males and females in the U.S. From this table, it becomes clear that the majority of patients are diagnosed with UBC at 60 years and older.

**Table 1:** Risks of developing urinary bladder cancer among US males and females (SEER data)

Age until/age from	10	20	30	40	50	60	70	80	90	Lifetime risk
Males										
0	0.00	0.00	0.01	0.02	0.09	0.35	1.06	2.35	3.53	3.86
10		0.00	0.01	0.02	0.09	0.35	1.07	2.38	3.57	3.90
20			0.00	0.02	0.09	0.35	1.08	2.38	3.58	3.92
30				0.02	0.08	0.35	1.09	2.41	3.62	3.96
40					0.07	0.34	1.09	2.43	3.67	4.01
50						0.28	1.05	2.44	3.71	4.07
60							0.83	2.32	3.68	4.07
70								1.74	3.33	3.77
80									2.26	2.89
90										1.60
Females										
0	0.00	0.00	0.00	0.01	0.03	0.12	0.33	0.69	1.05	1.18
10		0.00	0.00	0.01	0.03	0.12	0.33	0.70	1.06	1.19
20			0.00	0.01	0.03	0.12	0.33	0.70	1.06	1.19
30				0.01	0.03	0.12	0.33	0.70	1.06	1.19
40					0.02	0.11	0.32	0.70	1.07	1.20
50						0.09	0.31	0.69	1.06	1.19
60							0.23	0.62	1.01	1.15
70								0.44	0.87	1.02
80									0.54	0.73
90										0.38

This table shows the risk in % from a certain age (first column) until a certain age (top row). The highlighted cells contain the risk of developing bladder cancer in the next 10 years (also shown in the solid bars in Fig. 2). The last column contains the risks of cancer in the remaining lifetime from a certain age onwards (also shown in the dashed bars in Fig. 2)

## UBC mortality is less variable around the globe

**Fig. 3:**
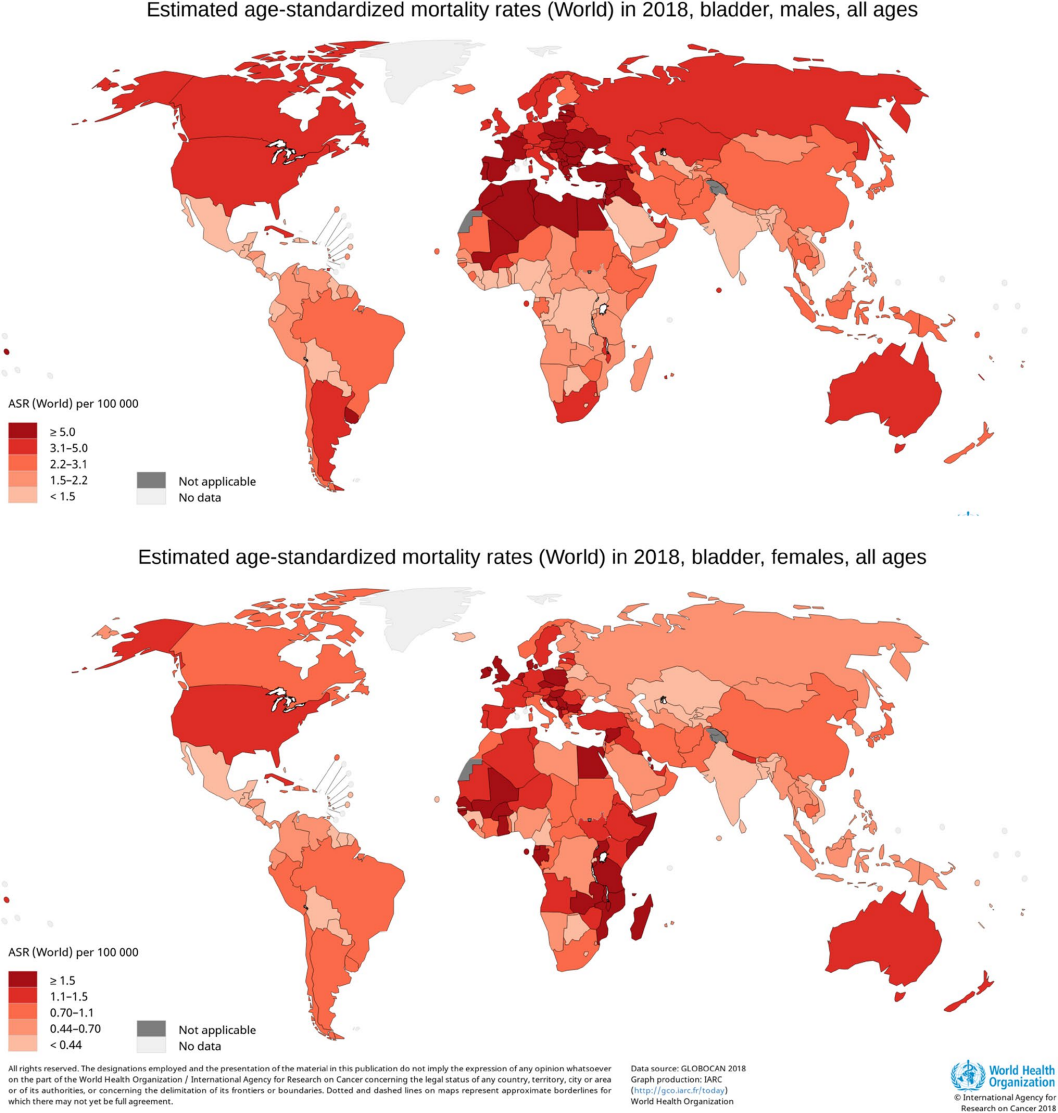
Urinary bladder cancer mortality across the world in 2018, by sex

Worldwide approximately 200,000 patients died from UBC in 2018 [6]. The global ASR for mortality among males is 3.2 per 100,000 per year versus 0.9 per 100,000 per year among females. The variability in UBC mortality around the world is not as large as that in UBC incidence. The ASR (per 100,000 person-years) among males varies between 4.3 in countries with a very high Human Development Index (HDI) and 2.0 in countries with a low HDI. For females, the ASRs per 100,000 person-years vary between 0.33 and 1.1. Figure 3 shows the ASRs for mortality for the world. The smaller variability is partly because there are less definition differences with respect to ≥ T2 cancers which are responsible for most of UBC mortality and are more often adequately registered as cause of death. Nevertheless, accuracy of causes of death registries may vary substantially [13].

## Five-year relative survival for UBC is around 70% in Europe

**Fig. 4:**
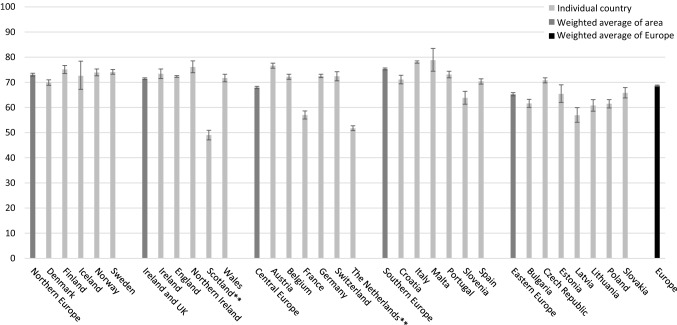
Five-year relative survival of urinary bladder cancer patients in European countries by sex. Data from EUROCARE-5 based on 429,154 cases of bladder cancer (ICD-O-3 topography C67, including non-invasive tumors) diagnosed between 2000 and 2007 with follow-up until 2008 from 86 population-based cancer registries from 29 European countries [14]. Relative survival rates are age-standardized. Error bars represent 95% confidence limits. Survival estimates from Scotland and The Netherlands reflect only invasive bladder cancer

Accurate population-based survival data are scarce, especially for less developed areas. From the EUROCARE-5 study, relative survival estimates for UBC patients in Europe are available and shown in Fig. 4. In Europe, the 5-year age-standardized relative survival rate of all UBC was approximately 70%, ranging from on average 60–80% between individual countries [14]. This enormous variation is probably partially explained by differences in proportions of non-invasive tumors. Although these data refer to patients diagnosed between 2000–2007, the study shows no to only minor improvement in survival rates over the years, which is corroborated in more recent data from SEER and the NCR.

In most countries, survival rates for women were slightly lower than for men. This cannot be completely explained by a diagnostic delay in women because stage-adjusted survival figures show the same difference [15]. The underlying mechanisms are multifactorial, probably including the thinner bladder wall in women and hormonal differences between genders. Recent studies from Norway and the Netherlands show that the survival difference between men and women only exist in the first 2 years of follow-up [16, 17].

It is likely that survival rates vary more between countries with different HDI levels, given less accessibility to optimal diagnostics and treatments and divergent competing risks of other causes of death. Because of the latter point, UBC-specific survival (or alternatively, relative survival) is preferable to overall survival to compare countries.

## 1.65 million people live with UBC worldwide

Prevalence, which is the number of patients alive with UBC at a specific point in time is a function of incidence and ‘duration of the disease’. It can be calculated by prevalence = incidence × duration. Duration is, of course, related to survival but it is also related to how long a patient with a history of UBC is considered to be a UBC patient. For that reason, the so-called ‘partial prevalence’ refers to the number of patients still alive within a defined period following diagnosis (usually 5 or 10 years).

**Fig. 5:**
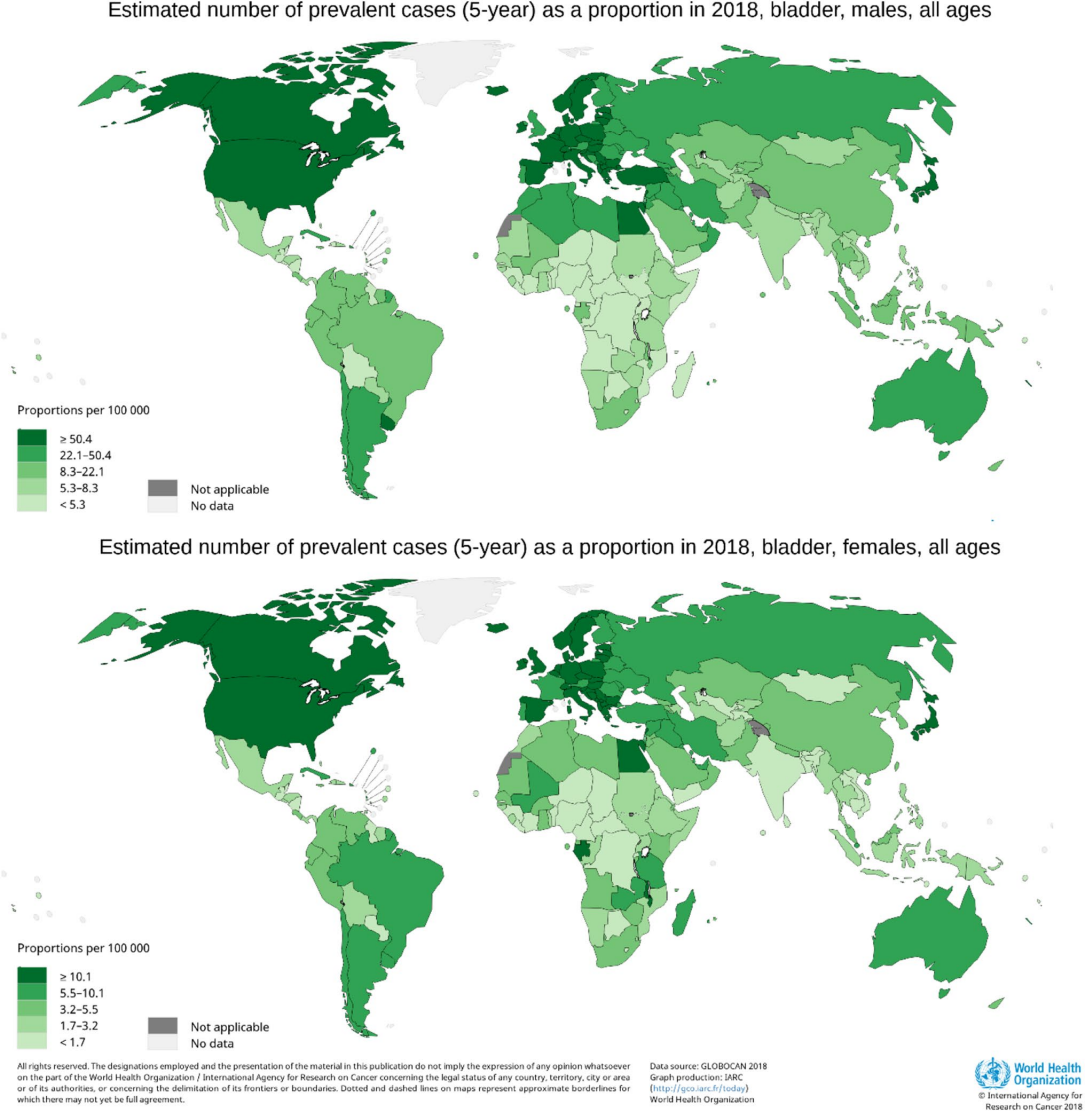
Five-year urinary bladder cancer prevalence rates in 2018 in the World, by sex

The overall global five-year prevalence is estimated at 1,650,000 (approximately 1,300,000 males and 350,000 females) in 2018 [6]. In most European and North-American countries the 5-year prevalence for men and women is over 50 per 100,000 and 10 per 100,000 persons, respectively (Fig. 5). Probably because of a lower incidence and worse survival, the prevalence is up to tenfold lower in countries such as Mexico, India, Mongolia and sub-Saharan African countries.

## Population growth and aging will increase the number of diagnoses, not the risk of UBC

According to the United Nations, the world population is expected to increase from the current 7.6 billion to 8.5 billion people in 2030 and passing 9.7 billion in 2050; for a large part caused by an expected doubling of the African population between now and 2050 [18].

Currently, people > 60 years old represent approximately 13% of the world population, varying from 25% in Europe to 5% in Africa. Half of the increase in world population will be reflected in a rise in the population aged > 60 years, rising from 960 million to 1.4 billion by 2030 and 2.1 billion by 2050 [18].

These demographic changes will have an enormous impact on UBC occurrence (as well as on most other late-onset diseases), and therefore also on prevalence and mortality. This will lead to an increasing burden on clinical care and society as a whole. It is good to realize that the risk of UBC until a certain age is not affected by such changes.

## Besides aging, tobacco smoking is the strongest determinant for the burden of UBC

The population attributable risk (PAR) of tobacco smoking for UBC has been estimated around 50% [19]. Hence, alterations in tobacco smoking prevalence will have considerable impact on incidence of UBC, although with a delay of several decades.

As of 2015, it seems like the tobacco epidemic is showing a decline in most countries in the world.

**Fig. 6:**
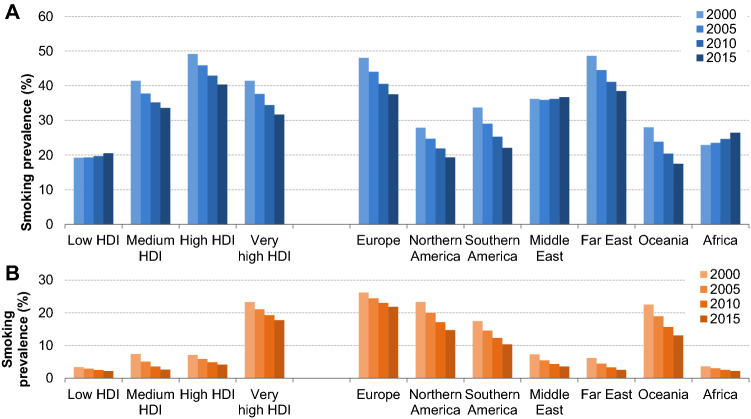
Smoking prevalence trends over time by sex and HDI/world region in **a** men and **b** women. Smoking defined as daily or occasional tobacco smoking. Smoking prevalence by sex was extracted from the Global Health Observatory data repository of the World Health Organization [34]. Human Development Index (HDI) per country was derived from the listing of the United Nations Development Programme’s Human Development Report of 2018 based on data of 2017. A weighted average across countries per HDI level/world region was calculated based on the population size of each country derived from the United Nations Population Division of 2018

Figure 6 shows that across medium, high and very high HDI countries there is a decreasing trend in tobacco smoking in both men and women. In the low HDI countries however, where the proportions of tobacco smokers are still much lower than in other HDI groups, it shows a small rise of tobacco use among men only. The rise in tobacco prevalence is mostly found in Africa (and partly the Middle Eastern countries), and only in men (Fig. 6). Provided that Africa is also the region that accounts for most of the world population growth, this trend is likely to have considerable impact on incidence.

It should be taken into account that the consequences of tobacco use manifest several decades later.

Smoking prevalence in Western Europe started to decline from the 1950s among men and from the mid 1970s among women [20]. Due to strong anti-smoking policies in the highest HDI countries, the decrease in UBC incidence is likely to continue, whereas an increasing trend is expected in low HDI countries lacking such policies.

Several policy actions in Western countries, such as high taxes on tobacco products and banning of tobacco advertising, have led to a considerable decrease of tobacco use. For instance, the prevalence of daily smoking among adult Australians is only 14.5%, in Norway 13.0% [21]. These policies, if implemented in other regions of the world, might help to mitigate the impact of the tobacco epidemic elsewhere. For instance, it could lower the overall level and duration of the tobacco epidemic in Africa and the Middle East and speed up the decrease in tobacco use in the Far Eastern countries, where such regulations are still sub-optimal despite recent improvements [22]. On the other hand, the tobacco industry is marketing its products more aggressively in low and middle income countries now that the fight against smoking is more prominent in the high income countries.

## Policy-level actions to cope with the global burden of UBC

This overview illustrates that the global burden of UBC is high, with an estimated 550,000 new cases of UBC every year. To date the burden is still highest in developed communities but with increasing exposure to risk factors, such as smoking, and a strongly increasing life expectancy, this burden is expected to increase in the developing world in the near future.

A major part of the future increase in UBC burden will be inevitably attributable to the growing and aging world population. The single major actionable contributor is still tobacco use, and part of the UBC burden can be averted with policy measures to prevent and stop tobacco use. Urgent action on cancer control should be taken with a focus on primary prevention of smoking in developing areas, especially in the Middle East and Africa. Smoking cessation should also be stimulated through policy measures, because it impacts overall life expectancy and quality of life and potentially also impacts the risk of recurrence and disease progression in UBC patients [23–25]. Smoking cessation also impacts the social surrounding of the ex-smoker, denormalizing smoking in society for other potential smokers [26].

The WHO Framework Convention on Tobacco Control is actively enforcing policies across the world to do so [27]. Policies that have been most successfully implemented so far are protection from tobacco smoke, packaging and labeling of products and sales to minors. Enforcement of tax and price measures on tobacco products is also increasing to suppress the demand for tobacco products, although this is lagging behind in other continents than Australia, Europe, and North America.

It is vital to curb the projected increase in UBC incidence because it affects population health and wellbeing, but not in the last place to control related health care costs. UBC has the highest lifetime treatment costs per patient of all cancers, with total costs of 3.6 billion euro per year in the US and almost 5 billion euro in Europe [28, 29]. This is due to high recurrence rates in the large group of non-muscle-invasive UBC patients, and associated intensive surveillance strategies, and expensive treatment costs [30]. Efforts to efficiently select patients with non-muscle-invasive UBC that will benefit from less intensive surveillance schedules will both mitigate health care costs of surveillance and burden on the individual patient. There have been several studies on the value of population-based screening for UBC but formal screening programmes have never been adopted [31]. In some occupational situations in which workers were exposed to bladder cancer carcinogens screening programmes have existed or may still exist [32]. In any case, the quantitative effect on UBC occurrence of screening for UBC seems very small. Another projected source of high expense is the use of expensive therapies in the final stages of disease (and soon in the neoadjuvant setting) with the recent introduction and future expanding use of checkpoint inhibitors. Research is necessary to identify patients most likely to benefit from these interventions. To make sufficient progress, funding for research focusing on UBC has to become more in line with the incidence and financial burden respective to other research priorities after years of disproportionate allocation [33].

## Conclusions

In conclusion, two demographic trends are largely responsible for increasing UBC occurrence worldwide: overall population growth and aging of populations (mostly on the African continent). Although a decreasing trend in tobacco use is observed in many areas of the world, this will only partly offset the increasing occurrence caused by demographic trends.

With increasing levels of development in traditionally lesser developed areas, better health care facilities will become available, which is expected to increase survival and subsequently prevalence. Efforts to reduce the overall worldwide burden of UBC include proceeding and scaling-up policies to decrease tobacco smoking, developing minimally intensive follow-up programs for patients with NMIBC, making high-quality facilities for diagnosis and management accessible in less-developed countries and developing ways to accurately identify patients who will benefit from expensive therapies.
